# Secondary metabolites in fungus-plant interactions

**DOI:** 10.3389/fpls.2015.00573

**Published:** 2015-08-06

**Authors:** Tünde Pusztahelyi, Imre J. Holb, István Pócsi

**Affiliations:** ^1^Central Laboratory, Faculty of Agricultural and Food Sciences and Environmental Management, University of DebrecenDebrecen, Hungary; ^2^Faculty of Agricultural and Food Sciences and Environmental Management, Institute of Horticulture, University of DebrecenDebrecen, Hungary; ^3^Department of Plant Pathology, Centre for Agricultural Research, Plant Protection Institute, Hungarian Academy of SciencesDebrecen, Hungary; ^4^Department of Biotechnology and Microbiology, Faculty of Science and Technology, University of DebrecenDebrecen, Hungary

**Keywords:** host-pathogen interaction, phytotoxin, phytoalexin, secondary metabolite, mycotoxin

## Abstract

Fungi and plants are rich sources of thousands of secondary metabolites. The genetically coded possibilities for secondary metabolite production, the stimuli of the production, and the special phytotoxins basically determine the microscopic fungi-host plant interactions and the pathogenic lifestyle of fungi. The review introduces plant secondary metabolites usually with antifungal effect as well as the importance of signaling molecules in induced systemic resistance and systemic acquired resistance processes. The review also concerns the mimicking of plant effector molecules like auxins, gibberellins and abscisic acid by fungal secondary metabolites that modulate plant growth or even can subvert the plant defense responses such as programmed cell death to gain nutrients for fungal growth and colonization. It also looks through the special secondary metabolite production and host selective toxins of some significant fungal pathogens and the plant response in form of phytoalexin production. New results coming from genome and transcriptional analyses in context of selected fungal pathogens and their hosts are also discussed.

## Introduction

Phytopathogenic fungi that are basically classified as necrotrophs, hemibiotrophs and biotrophs constitute one of the main infectious agents in plants, causing alterations during developmental stages including post-harvest, gaining nutrients from the plants they invade and, therefore, resulting in huge economic damage. Plants and fungi are rich sources of thousands of secondary metabolites (SMs), which consist of low-molecular weight compounds (the number of the described compounds exceeds 100,000) that are usually regarded as not essential for life while their role are quite versatile (Perez-Nadales et al., 2014; Scharf et al., 2014). Here, our primary aims were to overview the fungal-plant interactions and summarize special SM productions (e.g., phytotoxins and phytoalexins) in context of these interactions. Furthermore, the review also considers data from new fungal genome and transcriptome analyses. These data have hypothesized the biosynthesis of a much wider spectrum of SMs than we have actually isolated and characterized, and which could have strong effect on crop quality. In addition, these data proposed more possible physiological activities for the SMs than we thought before.

## Phytopathogenic fungi

### Lifestyle of phytopathogenic fungi

While the initial phases of pathogenesis do not differ fundamentally between necrotrophs, hemibiotrophs and obligate biotrophic fungi, different strategies are used to acquire nutrients. Necrotrophic fungi have broader host ranges than biotrophs and often enlist cell-wall-degrading enzymes and toxins, which can be small peptides or SMs (Howlett, 2006). In contrast to necrotrophic and hemibiotrophic fungal pathogens, obligate biotrophs are entirely dependent on living plant tissue and characterized by a number of sophisticated infection structures including appressoria, penetration hyphae and infection hyphae allowing the invader to suppress plant defense responses and to gain excess to host nutrients (reviewed by Mendgen and Hahn, 2002; Schulze-Lefert and Panstruga, 2003). Biotrophs establish haustoria for nutrient uptake (Panstruga, 2003), suppress induction of host defense and reprogram metabolism (Biemelt and Sonnewald, 2006). Biotrophic fungi and their metabolism has been studied on nonobligate biotrophs, such as *Cladosporium fulvum* (Thomma et al., 2005), *Magnaporthe grisea* (Talbot, 2003) and *Mycosphaerella graminicola* (Palmer and Skinner, 2002; Deller et al., 2011). Much less is known about the obligate biotrophs, such as powdery mildews or rust fungi. However, it appears that biotrophy is associated with a convergent loss of secondary metabolic enzymes and reduction in genes encoding specific transporters of toxin secretion and extrusion of host defense compounds usual in necrotrophic fungi. Nevertheless, the infection strategy of necrotrophic fungi is less complex than that of obligate biotrophs. Appressoria formed by typical necrotrophs such as *Cercospora, Ramularia, Rhynchosporium, Alternaria, Fusarium, Botrytis, Helminthosporium, Sclerotinia*, or *Verticillium* species, are inconspicuous, and infection hyphae formed within the host are quite uniform (reviewed by Horbach et al., 2011). Condon et al. (2013) suggested that, while necrotrophs and hemibiotrophs employ fundamentally contrasting mechanisms of promoting disease, the tools they utilize e.g., host-selective toxins (HST) and protein effectors basically overlap.

It cannot be forgot that there are numerous examples of fungi associated with plants as symptomless endophytes (e.g., black Aspergilli, Penicillia). However, in association with host plants, the symptomless endophytes have the capacity to either develop as pathogens or saprophytes, and in either state can become producers of mycotoxins (Palencia et al., 2010), rich sources of effector molecules.

### Fungal secondary metabolites

Fungal SMs can be divided into four main chemical classes: polyketides, terpenoids, shikimic acid derived compounds, and non-ribosomal peptides. Moreover, hybrid metabolites composed of moieties from different classes are common, as in the meroterpenoids, which are fusions between terpenes and polyketides. Analysis of available fungal genomes revealed that ascomycetes have more genes of secondary metabolism than basidiomycetes, archeo-ascomycetes, and chytridiomycetes, whereas hemi-ascomycetes and zygomycetes have none (Collemare et al., 2008). Ascomycete genomes code for on average 16 polyketide synthases (PKS), 10 non-ribosomal protein synthases (NRPS), two tryptophan synthetases (TS), and two dimethylallyl tryptophan synthetases (DMATS) with crucial importance in SM synthesis. These types of SM genes encode signature enzymes that can be enriched in secondary metabolism gene clusters and responsible for main synthesis steps of metabolites. PKS–NRPSs have been identified only in ascomycetes, with an average of three genes per species. *Neurospora crassa* as well as human pathogens *Coccidioides* spp. and *Histoplasma capsulatum* have a lower number of PKSs (1–9 genes), NRPS (3–6 genes) and PKS-NRPSs (0–2 genes) than other ascomycetes. High number of fungal species have more than 40 genes encoding PKS, NRPS, hybrids, TS, and DMATS in their genome, including *M. grisea* (45 genes) (Collemare et al., 2008) (Table 1). Synthesis of siderophores, a class of SMs for iron uptake also involves a NRPS that is also very important for the virulence of several fungi (e.g., *Cochliobolus heterostrophus, C. miyabeanus, F. graminearum*, and *A. brassicicola*) (Oide et al., 2006).

**Table 1:** **Distribution of secondary metabolite gene families in selected pathogenic fungi**.

**Species**	**PKS^a^**	**PKS-like**	**NRPS^b^**	**NRPS-like**	**Hybrid^c^**	**DMAT^d^**	**Total**	**References**
*A. alternata*	10	n.d.^e^	n.d.	n.d.	n.d.	n.d.	~10	Saha et al., 2012
*A. arborescens*	29	n.d.	5	n.d.	2	n.d.	~36	Hu et al., 2012
*C. fulvum*	10	n.d.	10	n.d.	2	1	~23	de Wit et al., 2012
*C. lunatus* CX-3	16	1	6	10	2	1	36	Gao et al., 2014
*C. lunatus* m118	14	1	5	9	2	2	33	Gao et al., 2014
*C. heterostrophus* C5	22	3	9	7	0	3	44	Gao et al., 2014
*C. zea-maydis*	11	2	7	8	1	1	30	Gao et al., 2014
*P. nodorum*	12	9	9	5	1	2	38	Gao et al., 2014
*P. tritici-repentis*	14	6	12	6	1	0	39	Gao et al., 2014
*P. teres f. teres*	18	1	27	n.d.	2	1	~49	Amselem et al., 2011
*B. cinerea*	16	6	6	8	0	1	37	Islam et al., 2012
*S. sclerotiorum*	16	2	5	5	0	1	29	Islam et al., 2012
*M. grisea*	12	3	5	6	3	3	32	Islam et al., 2012
*M. oryzae*	23	2	8	6	5	3	47	Gao et al., 2014
*A. flavus*	25	3	18	14	2	8	70	Gao et al., 2014
*A. niger*	15	1	12	2	5	0	35	Amselem et al., 2011
*F. graminearum*	12	2	10	10	0	0	34	Gao et al., 2014
*S. turcica*	23	3	9	7	2	2	46	Gao et al., 2014
*M. phaseolina*	19	16	15	13	12	0	75	Islam et al., 2012

*Listed organisms: Alternaria alternata; Alternaria arborescens; Cladosporium fulvum; Cochliobolus lunatus CX-3; Cochliobolus lunatus m118; Cochliobolus heterostrophus C5; Cercospora zea-maydis; Phaeosphaeria nodorum; Pyrenophora tritici-repentis; Pyrenophora teres f. teres; Botrytis cinerea; Sclerotinia sclerotiorum; Magnaporthe grisea; Magnaporthe oryzae; Aspergillus flavus; Aspergillus niger; Fusarium graminearum; Setosphaeria turcica; Macrophomina phaseolina*.

a *polyketide synthase*.

b *non-ribosomal peptide synthase*.

c *PKS-NRPS hybrid*.

d *dimethylallyl tryptophan synthetase*.

e *not determined*.

**Figure 1:**
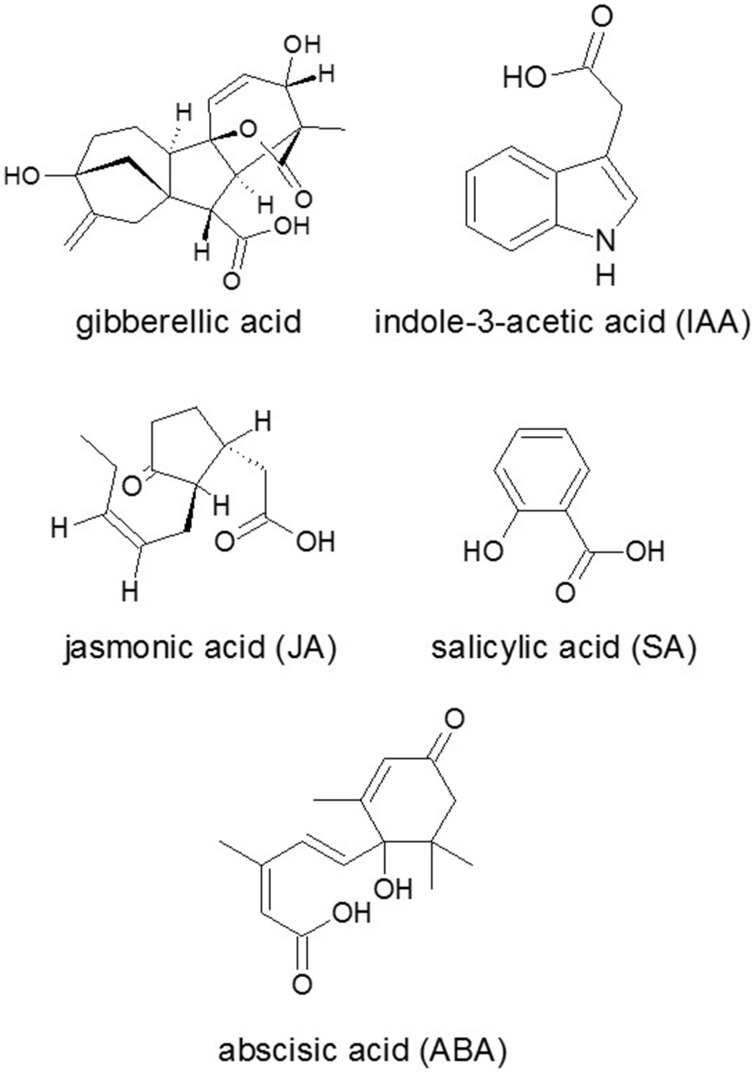
**Chemical structures of some plant hormones**. Source: National Center for Biotechnology Information. PubChem Compound Database (accessed Jun. 6, 2015) (Bolton et al., 2008).

Whole-genomic analysis have identified 12–15 PKS genes in *F. graminearum* (Kroken et al., 2003; Gaffoor and Trail, 2006; Gao et al., 2014; Sieber et al., 2014), where six have been linked to metabolites. The remaining PKSs have no assigned products yet even though they were expressed under tested conditions. In *F. graminearum*, the genes with known functions (13 SM genes) cover only a minor fraction of the 51 predicted SM genes: 15 PKSs, 19 NPSs and 17 TSs were identified (Sieber et al., 2014). Besides the classical SM genes (TS, NPS, and PKS) the 114 predicted genes encoding cytochrome P450 enzymes are also suitable candidates for searching SM gene clusters. Cytochrome P450s play an essential role in many known biosynthetic pathways of fungal compounds, for instance in the biosynthesis of trichothecene mycotoxins (Tokai et al., 2007) and gibberellins (Hedden et al., 2001) (Figure 1).

**Figure 2:**
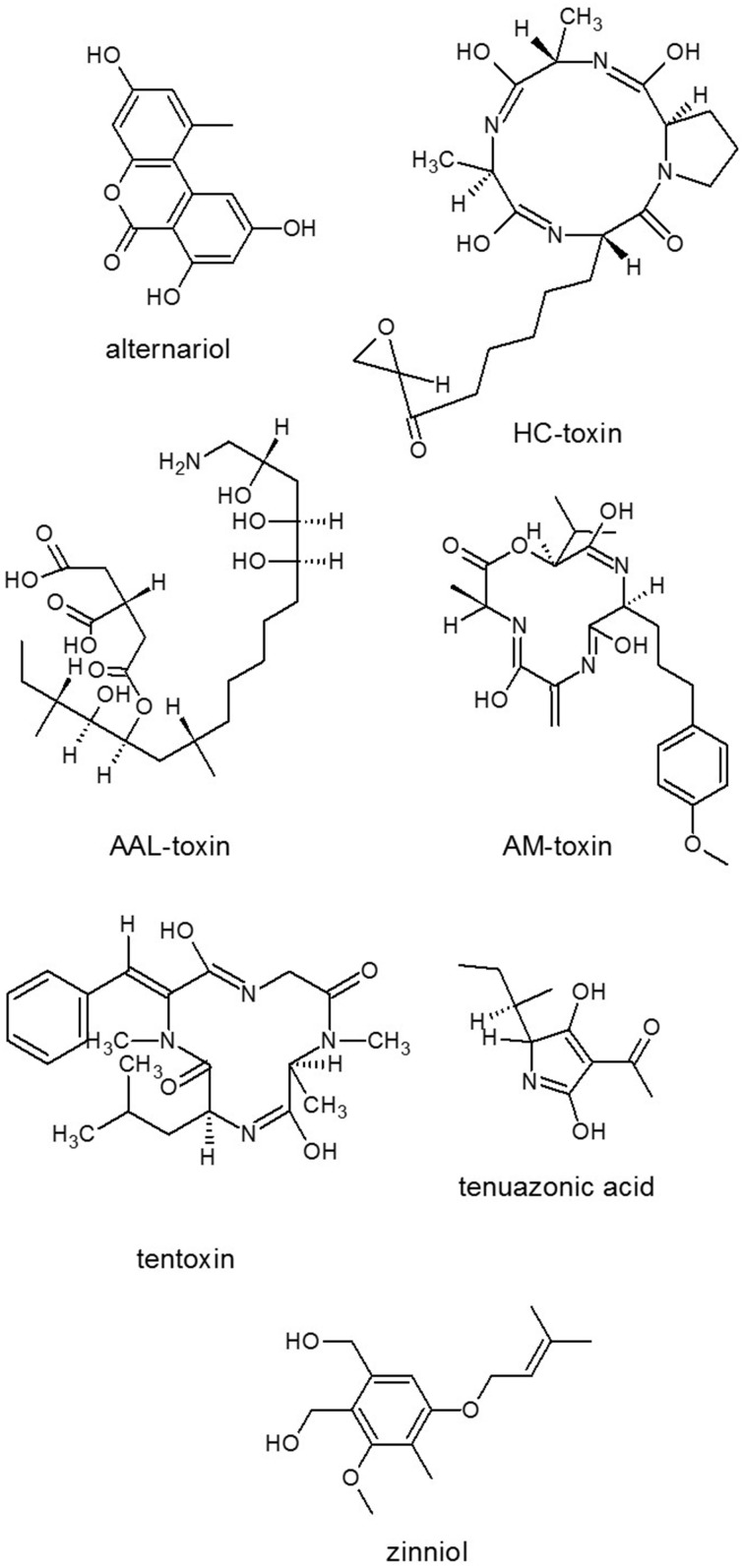
**Structures of representatives of ***Alternaria*** SMs**. Source: National Center for Biotechnology Information. PubChem Compound Database (accessed Jun. 6, 2015) (Bolton et al., 2008).

In the *Macrophomina phaseolina* anamorphic fungus from the ascomycete family *Botryosphaeriaceae*, Islam et al. (2012) identified 75 putative SM genes compared with 32 in *M. grisea*, 37 in *B. cinerea*, 29 in *S. sclerotiorum*, and 37 in *F. graminearum*. A high number of NRPSs which catalyze the production of cyclic peptides including numerous toxins were also found (Table 1). In *M. phaseolina* an NRPS, which showed 46% identity to *Cochliobolus carbonum* HST1, the key enzyme responsible for the biosynthesis of the maize HST cyclic tetrapeptide HC-toxin (Figure 2) (Panaccione, 1993; Walton, 2006). In 10 different *Fusarium* species including *F. graminearum, F. verticillioides, F. solani, F. culmorum, F. pseudograminearum, F. fujikuroi, F. acuminatum, F. avenaceum, F. equiseti*, and *F. oxysporum* comparative analyses of PKSs and NRPSs led to identification of 52 NRPSs and 52 PKSs orthology groups, respectively (Hansen et al., 2015). A core collection of eight NRPSs (NRPS2–4, 6, 10–13) and two PKSs (PKS3 and PKS7) were only conserved in the investigated strains. The genome of the saprophytic model organism *A. nidulans* contained 56 putative SM core genes including 27 PKS, two PKS-like, 11 NRPS, 15 NRPS-like genes, and one hybrid NRPS-PKS gene (Yaegashi et al., 2014).

The genome sequences of *B. cinerea* and *Sclerotinia sclerotiorum* were determined by Amselem et al. (2011). The *B. cinerea* genome showed high sequence identity and a similar arrangement of genes to *S. sclerotiorum*. The genomes contained a significant number of genes encoding key SM enzymes (Islam et al., 2012), however, the two fungi differed strikingly in the number and diversity of SM gene clusters, which may be involved in the adaptation to different ecological niches (Islam et al., 2012). These fungi had the potential to produce ~26 and 40 main SMs, respectively, as some SM pathways have more than one key enzyme (Amselem et al., 2011).

### Stimuli in fungal SM production

A high degree of environmental interaction, particularly sources of abiotic stress for either the host or the fungus such as drought or heat stress, also affect on the interactions (e.g., Fountain et al., 2014). Fungal genes involved in stress related responses, especially to oxidative stress, are highly represented in phytopathogenic fungi (see e.g., FSRD: Fungal Stress Response Database; Karányi et al., 2013) and fungal SM toxins often play a role in triggering these responses. Some fungal SMs, such as pigments, polyols and mycosporines, are associated with pathogenicity and/or fungal tolerance to several stress-inducing environmental factors, including temperature and UV light (Sinha et al., 2007). Moreover, environmental factors (e.g., light, temperature, pH, calcium, and nutrients) regulate SM production in a concerted way.

**Figure 3:**
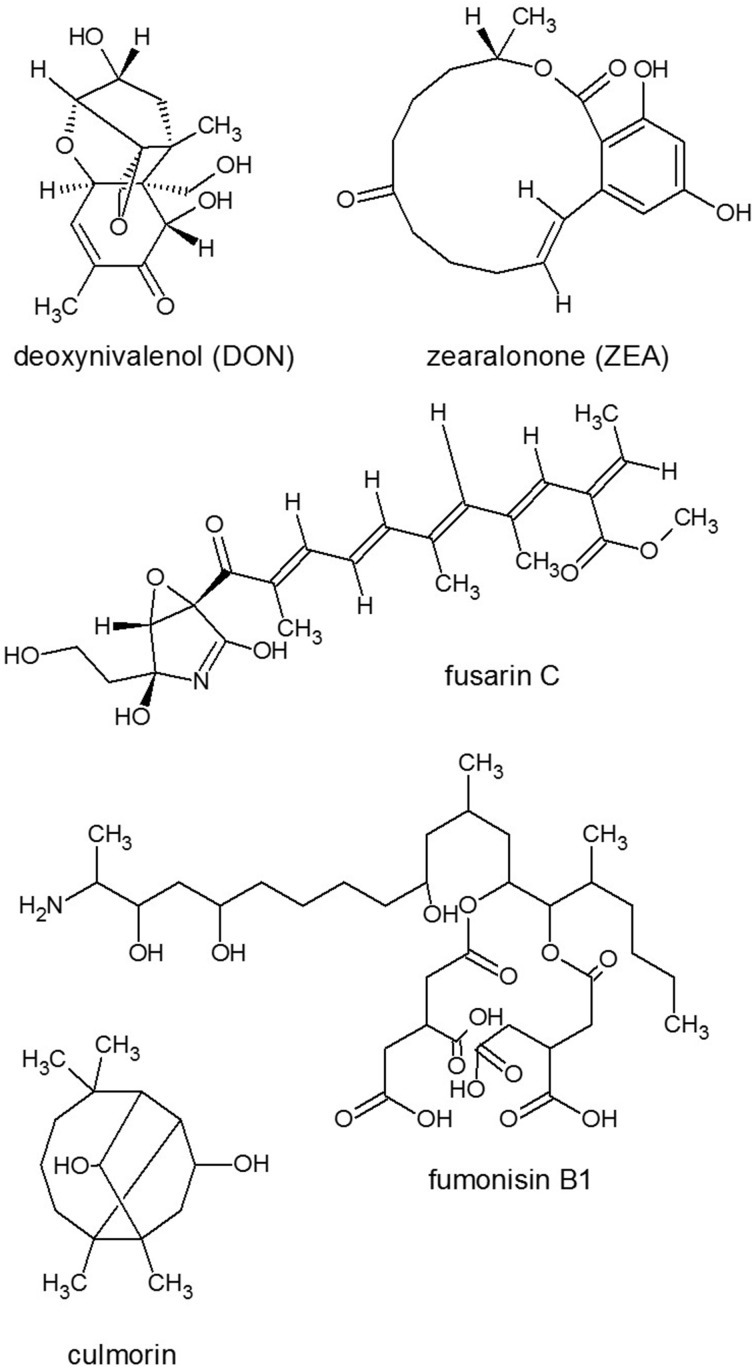
**Structures of representatives of ***Fusarium*** SMs**. Source: National Center for Biotechnology Information. PubChem Compound Database (accessed Jun. 6, 2015) (Bolton et al., 2008).

Light is a requirement for deoxynivalenol (DON) toxin (Figure 3) to exert its deleterious effect similarly to the induction of programmed cell death (PCD) during *Botrytis* infections (Govrin and Levine, 2002). This might reflect the plant's need for light to produce reactive oxygen during the oxidative burst (Howlett, 2006). Meanwhile, regulation of toxin production is also light-dependent (Avalos and Estrada, 2010) through one of the most important light-regulatory protein complex, the velvet complex, comprising at least *Fg*Ve1 and *Fg*VeB in *Fusarium* with homologous components in other fungi (Yang et al., 2013; Amare and Keller, 2014). *Fg*Ve1 homolog VeA has been demonstrated to regulate trichothecene production at the level of the biosynthetic genes *Tri4* and *Tri5* and the transcriptional regulator genes *Tri6* and *Tri10* (Jiang et al., 2011; Merhej et al., 2012). Disruption of VeB gene led to several phenotypic defects, including suppression of aerial hyphae formation, reduced hyphal hydrophobicity, highly increased conidiation and reduced DON biosynthesis through the regulation of *Tri5* and *Tri6* (Jiang et al., 2012). Deletion of LaeA (a nuclear regulator from the velvet complex) homolog Lae1 in *F. verticillioides* resulted in reduced expression of gene clusters responsible for synthesis of the SMs bikaverin, fumonisins (Figure 3), fusaric acid and fusarins (Figure 3). Analysis of SMs in the *F. verticillioides* LAE1 mutant revealed differences of regulation from that of in *F. fujikuroi* LAE1 mutant (Wiemann et al., 2010) as bikaverin production was reduced, but the amount of fumonisin B1 (FB1) (Figure 3) remained unchanged (Butchko et al., 2012).

**Figure 4:**
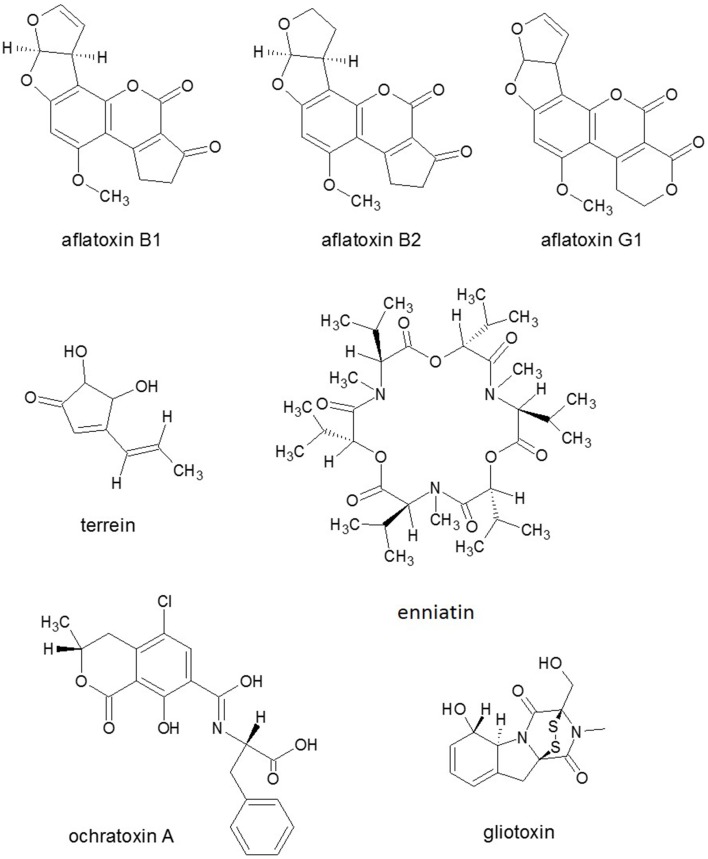
**Structures of representatives of ***Aspergillus*** SMs**. Source: National Center for Biotechnology Information. PubChem Compound Database (accessed Jun. 6, 2015) (Bolton et al., 2008).

Nitrogen limitation have appeared to be an essential stimulus for the activation of virulence functions in phytopathogenic fungi. The ability to metabolize a wide variety of nitrogen sources enables fungi to colonize different environmental niches and survive nutrient limitations (Tudzynski, 2014). Amino acids are required for SM biosynthesis, especially for the NRPS. Amino acid limitation in fungi results in the induction of a genetic network that induces genes for enzymes of multiple amino acid biosynthetic pathways as well as for aminoacyl-tRNA synthases. Inorganic N sources are also affect SM production. Ammonium activated the expression of aflatoxin (AF) (Figure 4) genes (Feng and Leonard, 1998), while nitrate served as an inhibitor of AF biosynthesis of *Aspergillus parasiticus* (Bagheri-Gavkosh et al., 2009). In all fungal species studied, the major GATA transcription factor AreA and its co-repressor Nmr were central players of the nitrogen regulatory network (Tudzynski, 2014). The importance of global nitrogen regulators for the development of pathogenicity was shown for *M. grisea* (Talbot et al., 1997) and many other fungal plant pathogens, e.g., *Colletotrichum lindemuthianum, C. acutatum*, and *F. oxysporum* (Kroll et al., 2014). In *F. graminearum*, which causes crop disease, nitrogen starvation activated the trichothecene pathway and induced the biosynthesis of the DON toxin (Figure 3) that was identified as a virulence factor (Desjardins et al., 1993; Audenaert et al., 2014), similar to the host selective T-toxin from *Cochliobolus heterostrophus* (*Bipolaris maydis*) (Turgeon and Baker, 2007) and the cyclic peptide AM-toxin (Figure 2) from *Alternaria alternata* (Markham and Hille, 2001).

Fungal toxin production is also regulated by signals or even substrates from plant. The well-characterized oxylipins (a group of diverse oxygenated polyunsaturated fatty acids) such as jasmonic acid (JA) (Figure 1) and its immediate precursor 12-oxo-phytodienoic acid are formed enzymatically in plants and accumulate in response to various stresses, in particular wounding and pathogen infection (Wasternack, 2007). These compounds are also formed non-enzymatically *via* the action of reactive oxygen species (ROS) (Wu and Ge, 2004), which also accumulate in response to pathogen infection, heavy metal uptake, or other stresses.

Fungal species have been shown to harbor or secrete JA and its derivatives (Miersch et al., 1999). Fungal oxylipins are able to mimic plant oxylipins; therefore, a reciprocal crosstalk was proposed between plant and fungus (Brodhagen et al., 2008), and several examples have proven this theory. The tomato-infecting *F. oxysporum* produced JAs using a lipoxygenase enzyme related to those found in plants, suggesting that JA biosynthesis in pathogenic fungi occurs *via* a pathway similar to that in plants (Brodhun et al., 2013). In *Aspergillus flavus*, oxylipins are molecules of quorum sensing. At low extracellular oxylipin concentration the cultures were characterized by increased sexual reproduction (sclerotia production), reduced conidiation (Horowitz Brown et al., 2008) and increased AF biosynthesis (Horowitz Brown et al., 2008; Affeldt et al., 2012; Amare and Keller, 2014). Moreover, deletion of oxylipin-encoding dioxygenase genes (*ppo* genes) of *A. flavus* resulted in decreased pathogenicity on host seeds. Exposure to the exogenous plant oxylipins 9(S)-hydroperoxyoctadecadienoic (9(S)-HpODE) acid and 13(S)-hydroperoxyoctadecadienoic acid (13(S)-HpODE) influenced positively the sporulation and effected precursor sterigmatocystin and AF synthesis in *A. flavus* as well as in *A. nidulans* and *A. parasiticus* (Calvo et al., 1999). In a lipidomic approach, Scarpari et al. (2014) have proven the important role of maize oxylipins in driving SM production in *A. flavus*; however, the mechanism of the action has been remained unsolved.

### Effects of phytotoxins on host plant

Fungal phytotoxins are usually divided into host-selective toxins (HSTs) and non-host selective (NHSTs) toxins. Typically, HSTs are active only toward host plants, have unique modes of action and toxicity to the host (Otani et al., 1995); moreover, the production of the HSTs is crucial for the virulence of these fungi (Walton, 1996; Horbach et al., 2011; Tsuge et al., 2013). Nearly all HSTs identified so far are produced by necrotrophic pathogens of the order of *Pleosporales* within the class of *Dothideomycetes* and especially in *Alternaria* and *Cochliobolus* species (Friesen et al., 2008; Stergiopoulos et al., 2013). These HST toxins are diverse chemically ranging from low-molecular-weight compounds to cyclic peptides. Genes encoding polypeptides for biosynthesis of these HSTs have been shown to reside on a conditionally dispensable chromosome that controls host-specific pathogenicity (Hatta et al., 2002). The mechanism of host-selective pathogenesis, through the HSTs, is well understood and about 20 HSTs have been documented (Otani et al., 1995; Walton, 1996). In some cases, host sensitivity was mediated by gene-for-gene interactions, and the toxin sensitivity was mandatory for disease development (Wolpert et al., 2002). Contrarily, NHSTs are not primary determinants of host range and not essential for pathogenicity, although they may contribute to virulence. These toxins have a broader range of activity, causing symptoms not only on hosts of the pathogenic fungus but also on other plant species (Walton, 1996).

**Figure 5:**
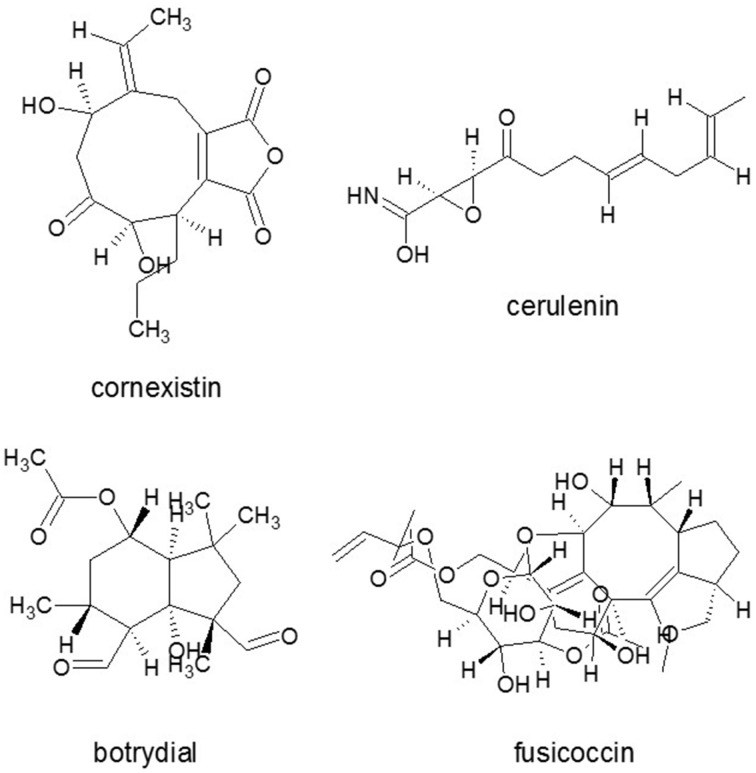
**Phytotoxic SM molecules from diverse fungi**. Cornexistin from *Paecilomyces variotii*, fusicoccin from *Fusicoccum* (*Phomopsis*) *amygdali*, cerulenin *Cephalosporium caerulens* and botrydial from *Botrytis cinerea*. Source: National Center for Biotechnology Information. PubChem Compound Database (accessed Jun. 6, 2015) (Bolton et al., 2008).

Several microbial phytotoxic compounds either inhibited an amino transferase or appeared to have such a mode of action, like cornexistin (Figure 5) from *Paecilomyces variotii* (Amagasa et al., 1994), which was patented as an herbicide; or tentoxin (Figure 2), a cyclic tetrapeptide from *A. alternata*, which indirectly inhibited the chloroplast development (Halloin et al., 1970). A series of structurally related fungal metabolites specifically inhibited ceramide synthase (sphinganine-*N*-acyltransferase) in plants, e.g., several analogs of AAL-toxin (*A. alternata*) (Figure 2) and FB1 (Figure 3) (*Fusarium* spp.) (e.g., Abbas et al., 1994). Fusicoccin (Figure 5) [*Fusicoccum* (*Phomopsis*) *amygdali*] irreversibly activated the plant plasma membrane H^+^-ATPase (Paiardini et al., 2014). Alternariol (Figure 2) and monomethyl alternariol are natural phytotoxins, produced by *Nimbya* and *Alternaria*, inhibited the electron transport chain (Demuner et al., 2013). Cerulenin (Figure 5) (*Cephalosporium cerulens*) inhibited *de novo* fatty acid synthesis in plastids (Laskay et al., 1985). T-toxin (a family of C35 to C49 polyketides) from *C. heterostrophus* (Levings et al., 1995; Inderbitzin et al., 2010), which is a HST trichothecene phytotoxin, inhibited mitochondrial respiration by binding to an inner mitochondrial membrane protein in sensitive plants, resulting in pore formation, leakage of NAD^+^, and other ions, as well as subsequent mitochondrial swelling (reviewed by Rocha et al., 2005). Zinniol (Figure 2) (*Alternaria* species and one *Phoma* species) bound plant protoplasts and stimulated Ca^2+^ entry into cells (Thuleau et al., 1988). The availability of fungal genome sequences, the knowledge of the biosynthesis of these toxins and gene disruption techniques, allows the development of tools for discovering the role of more and more toxins in plant cell death and disease.

## Secondary metabolite production on the host's side

Based on their biosynthetic origins, plant SMs can be divided into three major groups, (i) flavonoids and allied phenolic and polyphenolic compounds, (ii) terpenoids, (iii) nitrogen-containing alkaloids and sulfur-containing compounds, while other researchers have classified plant SMs into more specific groups (Wink, 2003) (Table 2). Plant SMs functions as defense molecules against microbes, viruses or other competing plants or as signal molecules like hormones and attracting molecules for pollinators or seed dispersal animals. Therefore, these compounds have importance for survival and fitness (Wink, 2003).

**Table 2:** **Classification of plant secondary metabolites**.

**Groups**	**Chemical structures**	**Examples**
Phenolics with one aromatic ring	C6	Phenol, hydroquinone, pyrogallol acid
	C6-C1	Gallic acid, salicylic acid, methyl syringate, vanillic acid
	C6-C2	Acetophenones, apocynin
	C6-C3	Hydroxycinnamic acid, ferulic acid, sinapic acid, coumaric acid, eugenol, zosteric acid
Phenolics with two aromatic rings	C6-C1-C6 Xanthones	Mangostin
	C6-C2-C6 Stylbenes	Reservatrol, chlorophorin
	C6-C3-C6 Flavonoids	Quercetin, glyceollin, sakuranetin
Quinones	Naphthoquinones Antraquinones Benzoquinones	Alizarin, emodin
Flavonoid polymers and non flavonoid polymers		Tannins
Terpenoids	C5 Hemiterpene	Isoprene, prenol, isovaleric acid
	C10 Monoterpene	Limonene, cineol, pinene, thymol, camphor, turpentin, carvacrol, citral, γ-terpinene, myrcene
	C15 Sesquiterpene	Abscisic acid, humulanes, culmorin, gossypol, zealexin
	C20 Diterpene	Gibberellin, taxol, oryzalexins, phytocassanes, momilactone, kauralexin
	C30 Triterpene	Brassinosteroids, squalen, lanosterol, avenacin
	C40 Tetraterpene	Carotenoids, lycopen
	C > 40 Polyterpenes	Rubber, glisoprenin
	Mixed origin (meroterpenes)	Cytokines, vitamine E
Nitrogen-containing	Alkaloides	Tomatin, solanin, nicotine
	Glucosinolates	Sinigrin, glucobrassicin
	Non protein amino acids	L-canavanine
	Amines	Phenylethylamine, tyramine, morphin
	Cyanogenic glycosides	Amygdalin, sambunigrin, linamarin

### Hormone production and plant resistance

Hormone biosynthetic pathways are typically involved in the regulation of plant resistance to pathogens and pests. Endogenous signaling molecules, e.g., ethylene (ET) (Ton et al., 2002), SA (Figure 1; Janda and Ruelland, 2014), JA (Figure 1; Wasternack, 2007; Van der Ent et al., 2009) and abscisic acid (ABA; Figure 1) (Hauser et al., 2011) have been associated with plant defense signaling against biotic stress. Generally, SA signaling induces defense against biotrophic pathogens, whereas JA against necrotrophic pathogens (Glazebrook, 2005).

**Figure 6:**
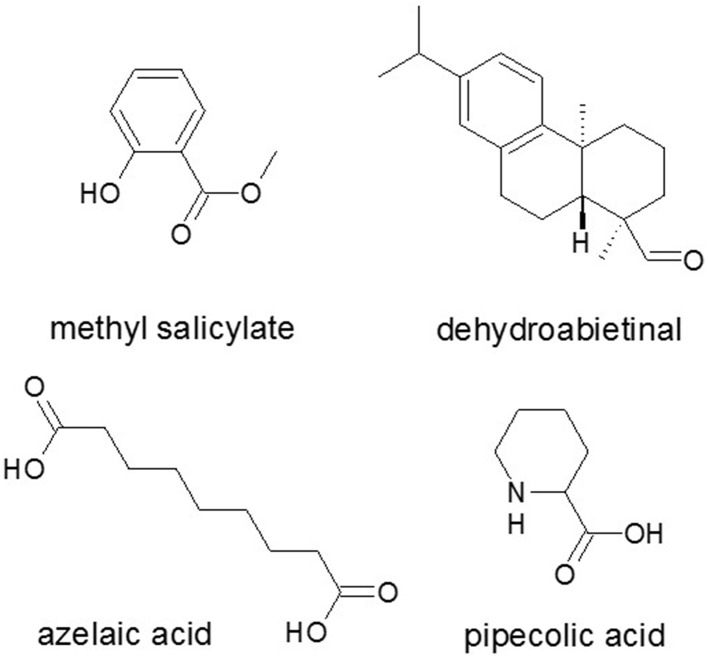
**Small metabolites as effectors in SAR signaling**. Methyl salicylate, the abietane diterpenoid dehydroabietinal, pipecolic acid from lysine catabolism, and the dicarboxylic acid azelaic acid are effectors in SAR signaling additionally to SA. Source: National Center for Biotechnology Information. PubChem Compound Database (accessed Jun. 6, 2015) (Bolton et al., 2008).

SA synthesis is a crucial way a plant responds to a biotic attack and involved in both local and systemic resistance (Janda and Ruelland, 2014). Systemic acquired resistance (SAR) is a plant immune response (Shah et al., 2014) that is induced after a local infection and confers immunity throughout the plant to a broad spectrum of pathogens. The onset of SAR (Durrant and Dong, 2004) is usually associated not only with increased levels of SA but additional small metabolites (Figure 6) have also been involved as effectors. Some of these metabolites have been implicated in the rapid activation of defenses in SAR in response to subsequent exposure to the pathogen that called priming (Shah et al., 2014).

The induced systemic resistance (ISR) pathway is stimulated during necrotrophic bacterial attack but was shown to protect *Arabidopsis* against the necrotrophic fungal pathogens *Alternaria brassicicola* (Ton et al., 2002), *Botrytis cinerea* (Van der Ent et al., 2008) and also *Plectosphaerella cucumerina* (Segarra et al., 2009), where SAR was ineffective (Van der Ent et al., 2009). Investigations of the regulation of ISR revealed the role of JA and ET (Ton et al., 2002; Yan et al., 2002; Kazan and Lyons, 2014). SAR and ISR were characterized by the coordinated activation of pathogenesis-related (PR) genes, many of which encode PR proteins with antimicrobial activity such as chitinases (Van Loon et al., 2006). Soluble chitin fragments released from fungal cell wall through the action of plant chitinases were found to serve as biotic elicitors of defense-related responses like phytoalexin synthesis in plants (Ren and West, 1992; Walker et al., 2003). ISR-related effect of methyl JA and SA was shown to activate also some defense enzymes (Derksen et al., 2013), which play a role to save plant cell wall and also raise the antioxidant capacity in plant cells (Yao and Tian, 2005).

The main auxin in higher plants, indole-3-acetic acid (IAA) (Figure 1), has profound effects on plant growth and development (Zhao, 2010). Only the free form of IAA and related compounds are considered to be active. The majority of produced auxin, however, is conjugated mainly to amino acids and sugars and thereby inactivated. IAA induces e.g., the production of expansins, the proteins whose function is to loosen the cell wall. But, the loose cell wall is more vulnerable to the invasion of different types of pathogens (Ludwig-Müller, 2011). Similarly to bacterial pathogens, hemibiotrophic or necrotrophic fungi produced IAA, manipulated plant growth and subverted plant defense responses such as PCD to provide nutrients for their growth and colonization (Ludwig-Müller, 2015). *Magnaporthe oryzae* secreted IAA in its biotrophic phase especially in the area of the infection hyphae (Tanaka et al., 2011) and, in turn, provoked rice to synthesize its own IAA at the infection sites (Li et al., 2013). However, it has not been elucidated yet whether IAA production is for the manipulation of the host plant or also for the fungus's own benefit. The activation of an auxin-inducible promoter by fungal IAA indicated that the host plant responds transcriptionally to the secreted auxin. The molecular processes that lead to plant disease and also the prospects for sustainable control were reviewed by Wilson and Talbot (2009). Treatment of *F. culmorum* infected barley with IAA resulted in a reduction of symptoms and yield losses, even though IAA did not inhibit the growth of the fungus *in vitro*. The results indicated increases in the gene regulation for defense-associated genes (Petti et al., 2012).

Plant gibberellins are important phytohormones promoting plant growth and fungi also synthesize gibberellins among other several important terpenes (Keller et al., 2005; Khan et al., 2011). However, higher plants and fungi have evolved their complex gibberellic acid (Figure 1) biosynthetic pathways convergently as it was indicated by the amino acid sequence homology analysis of the proteins in their biosythetic pathways (Hedden et al., 2001). Nevertheless, gibberellic acids produced as SMs in the rice-infecting *F. fujikuroi* were good examples of phytohormone mimics (Bömke and Tudzynski, 2009). Fungal gibberellins were involved in plant infection, e.g., as growth modulators like IAA, cytokinins, and ABA (Figure 1). Interestingly, other *Fusarium* species seem to have lost the ability to synthesize gibberellic acid, suggesting that this is an advantage for *F. fujikuroi* over other pathogens (Wiemann et al., 2013). *Aspergillus fumigatus* also produced gibberellins, and the role of this fungal species was also rectified by its regulatory effect on other phytohormones (ABA, SA, and JA) under stress condition (Khan et al., 2011).

### Plant secondary metabolites–antifungal compounds

Most of the SMs like phytocassanes (Koga et al., 1995) have been reported to have antifungal properties at least *in vitro*. The flavonoids and allied phenolics, e.g., coumarins, lignans, and polyphenolic compounds, including tannins and derived polyphenols form one major group of phytochemicals (reviewed by Crozier et al., 2008). These compounds or their precursors are present in high concentrations in leaves and the skin of fruits and are involved in important defense processes such as UV resistance, pigmentation, disease resistance, stimulation of nitrogen-fixing nodules (Pierpoint, 2000). Phenolic compounds (reviewed by Balasundram et al., 2006) are derivatives of the pentose phosphate, shikimate, and phenylpropanoid pathways in plants. These are known to alter microbial cell permeability and to interact with membrane proteins, which cause deformation in the structure and functionality of these proteins. These disadvantageous changes may lead to dysfunction and subsequent disruption of the membranes including the following events: (i) dissipation of the pH gradient and electrical potential components of the proton motive force, (ii) interference with the energy (ATP) generating and conservation system of the cell; (iii) inhibition of membrane-bound enzymes, and (iv) prevention of substrate utilization for energy production (De Oliveira et al., 2011; El-Mogy and Alsanius, 2012).

**Figure 7:**
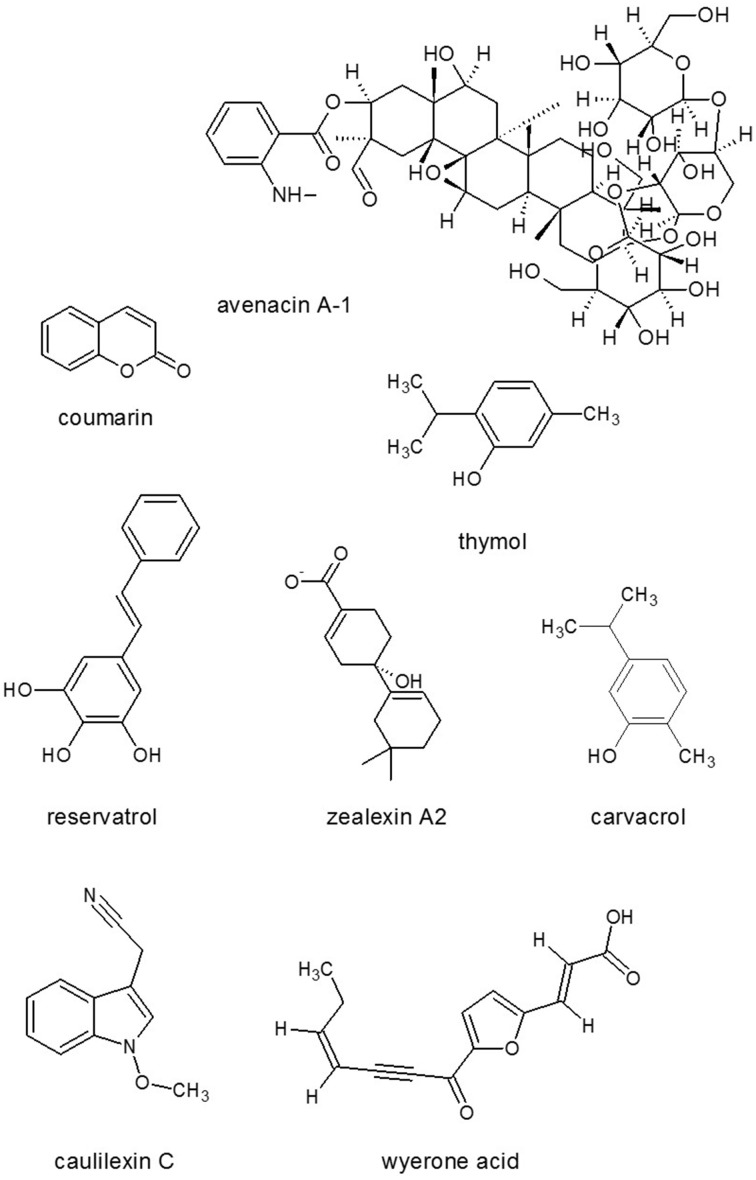
**Antimicrobials from plants**. Source: National Center for Biotechnology Information. PubChem Compound Database (accessed Jun. 6, 2015) (Bolton et al., 2008).

Antimicrobial compounds such as the steroidal glycoalkaloid saponins, e.g., avenacin (Figure 7) and α-tomatine, restrict the growth of pathogens in the apoplast. Saponins have strong antifungal activity; the tomato saponin α-tomatine activates phosphotyrosine kinase and monomeric G-protein signaling pathways leading to Ca^2+^ elevation and ROS burst by binding to cell membranes followed by leakage of cell components in *F. oxysporum* cells (Ito et al., 2007). Different plant species produce different types of saponins, which are effective against a wide range of pathogenic fungi (Osbourn, 1996). Terpenes are composed of several isoprene units, and can be linear or cyclic, and even saturated or unsaturated. The best-known terpenes are odoriferous plant metabolites like camphor and turpentine. The industrial and medical significances of plant terpenes, e.g., those of taxol, are reviewed by Bohlmann and Keeling (2008). In maize, sesquiterpenoid phytoalexins, zealexins (Figure 7), were discovered through characterization of physiological responses to the toxinogenic pathogen *F. graminearum*. Importantly, zealexins exhibited antifungal activity against several phytopathogenic fungi (*F. graminearum, A. flavus, Rhizopus microsporus*) at physiologically relevant concentrations (Huffaker et al., 2011).

**Figure 8:**
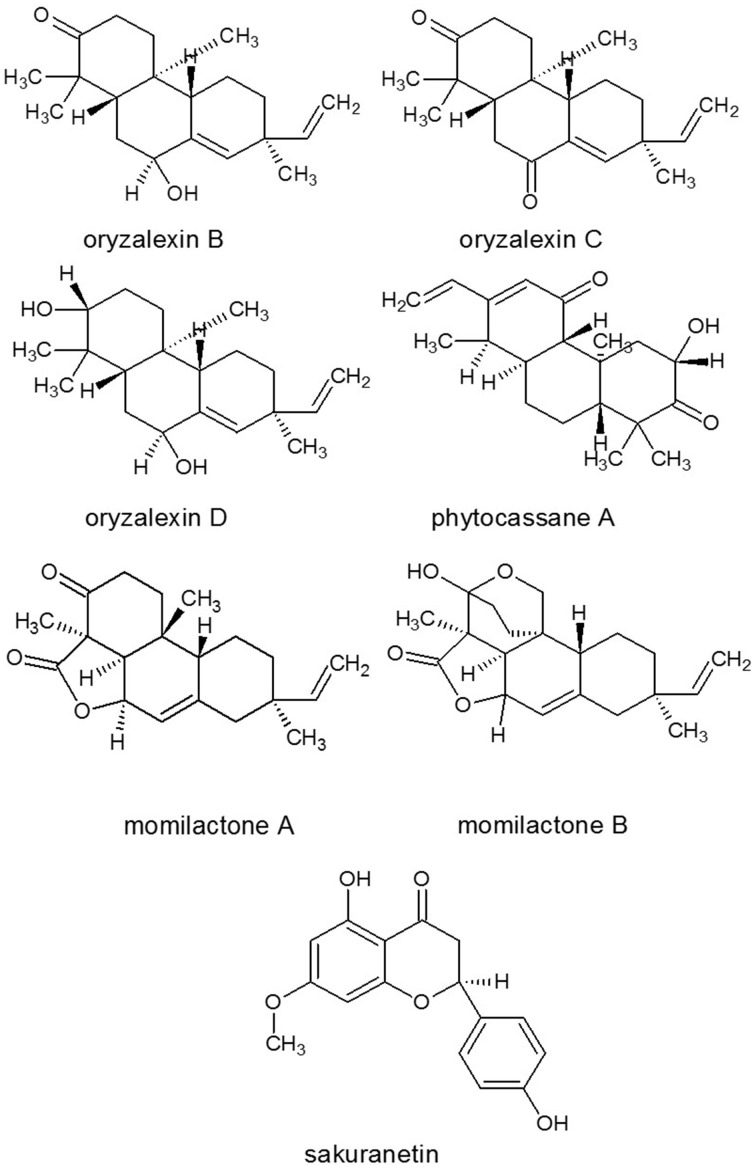
**Structures of some phytoalexins isolated from rice**. Source: National Center for Biotechnology Information. PubChem Compound Database (accessed Jun. 6, 2015) (Bolton et al., 2008).

Plant antifungal metabolites are preformed inhibitors that constitutively produced in healthy plants (phytoanticipins), or they may be synthesized *de novo* in response to pathogen attack or various non-biological stress factors such as short-wavelength UV light, treatment with heavy metal ions (e.g., copper or mercury salts). The letter pathogen and environmental stress elicited compounds are called phytoalexins. These groups cannot be separated strictly as the same compound may be a preformed antifungal substance in one species and can be phytoalexin in another. For example, flavanone sakuranetin (Figure 8) was found to be a phytoanticipin in *Ribes nigra* (Atkinson and Blakeman, 1982) and in *Hebe cupressoides* (Perry and Foster, 1994) but was induced in the leaves of rice *Oryza sativa* (Kodama et al., 1992). These metabolites can be constitutively present in one organ and can be induced in another. Plant SMs usually accumulate in smaller quantities than the primary metabolites (e.g., Dewick, 2002); however, they can accumulate in particular tissues (e.g., Takanashi et al., 2012) at a higher concentration. This accumulation is regulated in a highly sophisticated manner in appropriate compartments because some plant SMs are even toxic to the plants themselves if they are mislocalized. In the compartmentation and translocation processes, both primary and secondary transporters are involved and many transporter genes, especially genes belonging to the multidrug and toxin extrusion type transporter family, have been identified as responsible for the membrane transport of SMs (Yazaki, 2006; Yazaki et al., 2008). High number of SMs are well characterized in the families Fabaceae, Solanaceae and Labiaceae (Wink, 2003) as well as in cereals (reviewed by Du Fall and Solomon, 2011). Phytoalexins in families Fabaceae and Rosaceae and in rice were reviewed by Grayer and Kokubun (2001); while, SMs in a range of crop plants from families Cruciferae, Fabaceae, Solanaceae (Pedras and Ahiahonu, 2005), Brassicaceae, Vitaceae, and Poaceae (reviewed by Ahuja et al., 2012) have also been described recently.

Phyllosphere and rhizosphere microorganisms can live in a close mutualistic association with plants or even colonize plant tissues (endophytes). Plant growth-promoting non-pathogenic microorganisms like rhizobacteria and fungi are soil-borne microbes with beneficial effects on plant performance in the rhizosphere. They can stimulate plant growth by increasing tolerance to abiotic stress or by suppressing plant diseases (Van der Ent et al., 2009). Plants may actively shape microbial communities either inhabiting their outer surface or colonizing their interior (Bednarek et al., 2010). The growing plant secretes a wide range of chemicals, e.g., in root exudates, to communicate with rhizosphere microbes (Kolattukudy et al., 1995; Suryanarayanan et al., 2009; Baetz and Martinoia, 2014) such as arbuscular mycorrhiza. Altered exudation patterns, putative direct arbuscular mycorrhiza effects, different root size and architecture, altered physiology may contribute to quantitative and qualitative microbial community changes in the mycorrhizosphere caused by arbuscular mycorrhiza fungi (Wehner et al., 2010). Organic acids, amino acids and phenolic compounds present in root exudates play an active role in root-microbe communications (Dakora and Phillips, 2002; Tanimoto, 2005; Crowley and Kraemer, 2007; Li et al., 2013) and stimuli such as microbial elicitors trigger compositional changes in root exudates (Baetz and Martinoia, 2014). Walker et al. (2003) analyzed root exudates of *Arabidopsis thaliana* elicited by SA, JA and chitosan as well as by two fungal cell-wall elicitors and profiled the SMs subsequently secreted. Among the several compounds detected butanoic acid, trans-cinnamic acid, *o*-coumaric acid, *p*-coumaric acid, ferulic acid, *p*-hydroxybenzamide, methyl *p*-hydroxybenzoate, 3-indolepropanoic acid, gallic acid, and vanillic acid were successfully inhibited the growth of *F. oxysporum, Phytophthora drechsleri* and *Rhizoctonia solani* phytopathogenic fungi.

### Manipulation of programmed cell death

Different fungal strategies mediate killing of the plant host cells such as secretion of low molecular weight or peptide toxins or eliciting PCD in the host by secretion of ROS (Horbach et al., 2011; Barna et al., 2012). From the host's side, chloroplasts have a critical role in plant defense as these organelles are not only sites for the biosynthesis of the plant signaling compounds: SA, JA and nitric oxide but for ROS production as well (e.g., Lee et al., 2015). Therefore, chloroplasts are regarded as important players in the induction and regulation of PCD in response to both abiotic stresses and pathogen attack. Moreover, toxin effectors from necrotrophic fungi can target one of the host's central signaling/regulatory pathway to trigger resistance (R) gene-mediated resistance or to down-regulate defense enzymes, and, as a consequence, to increase thereby host susceptibility to fungal attack (Wang et al., 2014). *Aspergillus* mycotoxin ochratoxin A (Figure 4) induced necrotic lesions in detached leaves through oxidative burst induction with increased ROS level and concomitant down-regulation of plant antioxidant defense enzymes (Peng et al., 2010). SMs fusarenon, nivalenol, DON, T-2, HT-2, diacetoxyscirpenol, beauvericine and neosolaniol (Figure 3) from *Fusaria* caused complete inhibition of seed germination and induced PCD and alteration to ascorbate metabolism in tomato protoplasts (Paciolla et al., 2004). T-2 trichothecene toxin, produced by e.g., *F. sporotrichioides*, also induced cell death, callose deposition, generation of hydrogen peroxide, and accumulation of SA, while DON toxin inhibited translation without induction of the elicitor-like signaling pathway in the non-host plant *A. thaliana* (Nishiuchi et al., 2006). The ascomycete *Cochliobolus victoriae* is a necrotrophic fungal pathogen of *Arabidopsis* and oats with HST victorine, which induced defense-related responses such as phytoalexin synthesis, extracellular alkalization and PCD causing Victoria blight (Tada et al., 2005). Zhang et al. (2011) proposed that both JA and ET promote the *A. alternata* AAL toxin-induced PCD in detached *Solanum lycopersicum* leaves by disruption of sphingolipid metabolism (Spassieva et al., 2002). In *Arabidopsis*, free sphingoid bases were again shown to be involved in the control of PCD, presumably through the regulation of the ROS level upon receiving different developmental or environmental cues (Raffaele et al., 2009).

## Phytotoxins, phytoalexins and special SMs

### *Magnaporthe grisea* species complex

The *M. grisea* species complex comprises many phylogenetic species (Couch and Kohn, 2002) that cause disease to some 50 grass and sedge species. These include rice, wheat, barley, maize, oats, rye, finger millet, perennial ryegrass, weed and ornamental grasses. Within this species complex, *M. oryzae* (previously known as *M. grisea*) isolates form the pathotype *Oryza*, which causes rice blast disease. Approximately 10–30% of the annual rice harvest is lost due to the infection. The fungus infects all aerial parts of rice, leading to leaf blast, neck and panicle rot, collar rot and node blast (reviewed by Skamnioti and Gurr, 2009).

**Figure 9:**
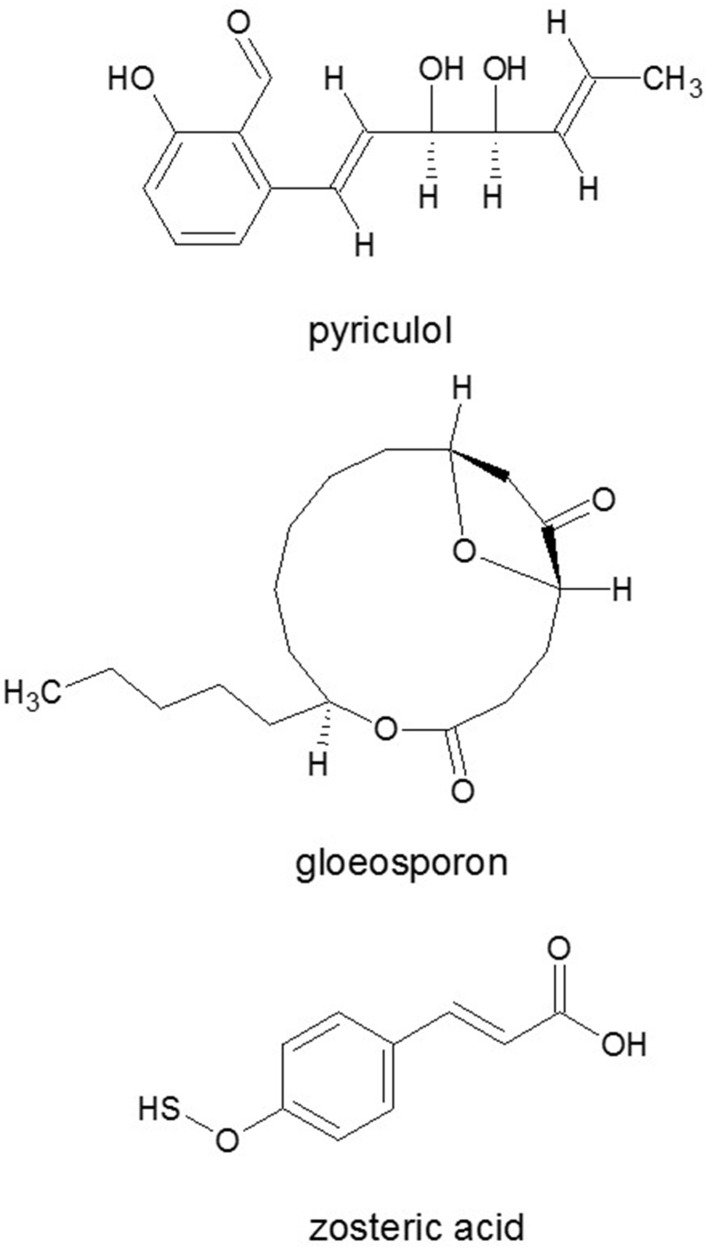
**Autoinhibitor signal molecules responsible for inhibition of conidia germination**. Phytotoxin pyriculol and gloeosporone, and/or appressorium formation: zosteric acid. Source: National Center for Biotechnology Information. PubChem Compound Database (accessed Jun. 6, 2015) (Bolton et al., 2008).

Chemical signals are responsible for appressorium formation in *M. grisea*. The appressorial glue of *M. grisea* contains glycoproteins, neutral lipids and glycolipids (Ebata et al., 1998). The non-toxic plant metabolite zosteric acid (Figure 9) (Todd et al., 1993) binds water and enhances the hydrophilicity of the surface, thereby weakening the binding capacity of the appressorial glue, which is highest with hydrophobic surfaces. Therefore, zosteric acid inhibits spore adhesion and infection by *M. grisea* and also by *Colletotrichum lindemuthianum* on artificial hydrophobic surfaces as well as on plant leaves (Stanley et al., 2002).

The two most effective inducers of the germination and appressorium formation were found to be 1,16-hexadecanedial and 1,16-hexadecanediol from cutin monomers in *M. grisea* (Gilbert et al., 1996). Besides cutin monomers, surface waxes also activated development processes in fungi (Liu et al., 2011). Appressorium formation was induced by leaf wax of rice or other plants or synthetic n-C22 fatty acid, fatty alcohol or alkane (Hegde and Kolattukudy, 1997). Self-inhibitors of the germination are related to phytotoxin pyriculol (Figure 9) (Kono et al., 1991). Moreover, other fungi can also produce specific, non-toxic inhibitors of conidial germination and appressorium formation of *M. grisea* like flaviolin, tenuazonic acid (Figure 2), and glisoprenins (Thines et al., 2004).

In the appressorium, several key biochemical and morphogenetic events take place under the generation of turgor pressure which, in *M. grisea*, is the highest pressure recorded in any living cell at up to 8 MPa to penetrate the tough rice cuticle (Howard et al., 1991). This exceptionally high pressure and mechanical penetration question the role of secreted cell wall-degrading enzymes in the first steps of invasion at least on the natural host (Howard and Valent, 1996). In order to generate the high turgor pressure, a thick melanin layer is deposited outside the primary cell wall. Several natural products inhibit melanin biosynthesis in a similarly specific and non-toxic manner, presumably hitting the same targets (Thines et al., 1995). Typical examples are coumarin (Figure 7), a common SM from plants (Wheeler and Bell, 1988), scytalol D from fungus *Scytalidium* sp. (Thines et al., 1998) and lipid biosynthesis inhibitor cerulenin (Figure 5) originally obtained from an isolate called *Cephalosporium caerulens* (Ohtake et al., 1999) that conspecific to *Sarocladium oryzae* phytopathogenic fungus of rice (Bills et al., 2004).

The first identified rice phytoalexins (reviewed by Peters, 2006) were the momilactones A and B (Figure 8) (Cartwright et al., 1981). Momilactones exhibit antifungal activity against *M. grisea* and only appear in rice leaves after infection (Kodama et al., 1988). These compounds were originally isolated and identified as plant growth inhibitors from rice seed (Kato-Noguchi et al., 2002). Another group of diterpenoid phytoalexins are oryzalexins (A-F) also isolated from rice (Akatsuka et al., 1985; Kato et al., 1993, 1994). Oryzalexins B, C and D (Figure 8) were identified as *ent*-pimarane diterpenoids, and found in *M. grisea* infected, but not healthy rice leaves (Akatsuka et al., 1985). Oryzalexin S (Tamogani et al., 1993), and phytocassanes A (Figure 8) to E (Koga et al., 1995, 1997) are also labdane-related diterpenoid phytoalexins in rice. Overexpression of another rice flavonon phytoalexin sakuranetin (Figure 8) resulted in an increased resistance to *M. grisea* (Kodama et al., 1992; Kim et al., 2009).

### Genus colletotrichum

*Colletotrichum* usually shares similar lifestyles and infection strategies with *M. grisea*, particularly during the early stages of pathogenesis. However, in hemibiotrophic *C. gloeosporioides* and in other *Colletotrichum* species unlike in the case of *M. grisea*, blocking the cell cycle did not prevent spore germination and appressoria formation (Nesher et al., 2008). The differentiation of infection structures including appressoria preceded mitosis and could proceed without nuclear division. Spore cell death did not occur during plant infection and the fungus primary infection structures remained viable throughout the infection cycle (Nesher et al., 2008).

The spores of many phytopathogenic fungi contain potent autoinhibitors, which prevent germination until they have been washed or diluted out of the spore. *Colletotrichum* spp. appeared to be a rich source of germination autoinhibitors. The first self-inhibitor to be isolated from conidia of *C. gloeosporioides* was gloeosporone (Figure 9) (Meyer et al., 1983) followed by (Z)- (E)-ethylidene-1,3-dihydroindole-2-one that were active at lower concentrations than gloeosporone (Tsurushima et al., 1995). At higher concentrations, the latter two compounds also inhibited the germination of conidia of other *Colletotrichum* spp. and of *F. oxysporum* (Tsurushima et al., 1995). Mycosporin-alanine is also a potent autoinhibitor of conidial germination in *C. graminicola* that was synthesized during the development of conidia in the pycnidium, and it was quite effective in preventing germination of the spores until they have become dispersed (Leite and Nicholson, 1992). Interestingly, the synthesis of mycosporines and mycosporine-like amino acids occurs in fungi, bacteria, cyanobacteria, phytoplankton and macroalgae but not in animals because it needs the shikimate pathway (Sinha et al., 2007). Conidia germination is induced by ET in ripened fruits (Flaishman and Kolattukudy, 1994), additionally, by fatty alcohols from cuticular waxes (Podila et al., 1993; Hwang and Kolattukudy, 1995).

### Botrytis cinerea

The gray mold fungus *B. cinerea* is a typical necrotrophic phytopathogenic fungus with a very wide host range. It causes vast economic damage pre- and postharvesting (Amselem et al., 2011). Two groups of its phytotoxic metabolites have been characterized, the sesquiterpene botrydial (Figure 5) and related compounds (Colmenares et al., 2002) and botcinic acid and its derivatives (Tani et al., 2006). The sesquiterpene-derived phytotoxin botrydial has been implicated in virulence, as it can be detected *in planta* and its addition facilitates fungal penetration and colonization of plants (Deighton et al., 2001). In addition to SM toxins, ROS play in important role in *B. cinerea* as the fungus actively contributes to the elevated levels of ROS detected at infection sites and causing an oxidative burst during cuticle penetration and lesion formation (Tiedemann, 1997; Tudzynski and Kokkelink, 2009).

After fungal attack of grapevine and berries, leaves produced phytoalexins such as resveratrol (trans-3,5,4′-trihydroxystilbene) (Figure 7) (Langcake and Pryce, 1976) and related compounds, which have antifungal activity toward *B. cinerea* and also a number of other fungal pathogens including *Rhizopus stolonifer* and *Plasmopara viticola* (Jeandet et al., 2002). Strong antifungal activity of carvacrol (Figure 7) and thymol (Figure 7) was also confirmed against *B. cinerea* and *R. solani, Fusarium moniliforme* and *S. sclerotiorum* (Mueller-Riebau et al., 1995; Tsao and Zhou, 2000; Camele et al., 2012). High inhibitory activity was detected against *B. cinerea* by monoterpene γ-terpinene (Espinosa-García and Langenheim, 1991), while monoterpene citral has been reported as a potent antimicrobial compound against *B. cinerea* (Tsao and Zhou, 2000) and *Penicillium italicum* (Saddiq and Khayyat, 2010). Essential plant oils (e.g., D-limonene, cineole, β-myrcene, α-pinene, β-pinene, and camphor) showed remarkably high antifungal activity against *B. cinerea* (Wilson et al., 1997). In *Vicia faba* tissues, low-molecular-weight phytoalexins such as wyerone acid (Figure 7) and wyerone furanoacetylenic were produced as part of the post-infection defense response against fungal pathogens. Wyerone acid accumulated in *B. cinerea* lesions, whereas in *Botrytis fabae* lesions the phytoalexin started to accumulate but later tended to decrease. The enhanced ability of *B. fabae* to colonize e.g., broad bean tissues seemed to be related to its capacity to detoxify broad bean phytoalexins (Buzi et al., 2003).

### Fusaria

The filamentous fungus *F. graminearum* (teleomorph: *Gibberella zeae*) is a worldwide pathogen of maize and small grains such as wheat, barley and oats. In infected grains, *F. graminearum* can produce several mycotoxins, including trichothecene derivatives (e.g., DON), polyketide zearalenone (ZEA), fusarin C (Figure 3) (Desjardins et al., 1993; Kimura et al., 2007) among which trichothecenes were related to the pathogenicity of *F. graminearum* (Gaffoor and Trail, 2006; Foroud and Eudes, 2009) reducing crop yield and quality. In plants, trichothecenes produced by *Fusarium* spp. cause necrosis, chlorosis, and mortality enabling them to mediate a wide variety of plant diseases, including wilts, stalk rot, root rot, and leaf rot in many important crop and ornamental plants (Abbas et al., 2013).

*F. verticillioides* (teleomorph: *Gibberella moniliformis*) is a ubiquitous pathogen of maize, attacking stalks, kernels, and seedlings. Considering the maize developmental stages, silking (R1), blister (R2), milk (R3), dough (R4), dent (R5), and physiological maturity (R6), infecting the seed at stages R2–R5 with *F. verticillioides* revealed that the pathogen colonized seeds equally well (Bluhm and Woloshuk, 2005). Nevertheless, significant sphingoid-derived fumonisin B1 (FB1) mycotoxin (Figure 3) production (Abbas et al., 1994; Bluhm and Woloshuk, 2005; Picot et al., 2011) occurred only in the R5 (dent)-stage kernels where the R5 kernel acidic state also induced more FB1 production (Picot et al., 2011). Expression of FUM8 and FUM12 fumonisin biosynthetic genes as well as low amounts of FB1 was detected in the R3 (milk) and R4 (dough) stages. In contrast, no FB1 or FUM gene expression was detectable in the R2 (blister) stage. Other experiments revealed that the fungus produced fourfold more FB1 on maize polysaccharide amylopectin than on glucose carbon source (Bluhm and Woloshuk, 2005; Picot et al., 2011).

Usually extracellular ATP functions as an endogenous external metabolite regulating plant cell viability. FB1 toxin could trigger the depletion of extracellular ATP, which altered the abundance of particular intracellular plant proteins and ended in cell death, which process was reversible by exogenous ATP (Chivasa et al., 2005). Nevertheless, FB1 did not appear to be a primary virulence factor, while DON (Figure 3) was considered to have a key role as a virulence factor at least in *F. graminearum*, and their induction is quite different. The production of DON and the spread of the fungus in the spikes correlated well with the presence of several polyamine compounds that accumulate as the infection progresses through the spike (Gardiner et al., 2010).

SM culmorins are tricyclic sesquiterpene diols that have been reported from *F. culmorum, F. graminearum* and *F. venenatum* (Langseth et al., 2001). Culmorin had weak phytotoxicity to wheat coleoptile tissue (Wang and Miller, 1988), but its role in wheat head scab was not reported. Contaminated grain samples are usually not screened for culmorins, because there are no limits for these SMs. However, culmorin and hydroxyculmorins were detected at relatively high levels in naturally contaminated Norwegian wheat, barley, and oat samples co-occurring with high DON concentrations (Ghebremeskel and Langseth, 2001).

Inhibition of toxigenesis in *Fusaria* has also been studied. Velluti et al. (2004) explored the efficacy of cinnamon, clove, lemongrass, oregano and palmarosa essential oils in order to prevent ZEA and DON accumulation when inoculated with *F. graminearum*; however, it should be noted that this assay was based on non-sterilized, naturally contaminated maize grain. Dambolena et al. (2011) studied the capacity of 10 natural phenolic compounds to inhibit FB1 synthesis by *F. verticillioides* and revealed that thymol, carvacrol (Figure 7), isoeugenol as well as eugenol were the most active. The plant phenol chlorophorin was also effective in reducing FB1 toxin production (94% reduction), followed by caffeic acid (hydroxycinnamic acid), ferulic acid, vanillic acid and iroko (Beekrum et al., 2003). In *F. proliferatum*, aquaeous extracts of host plants inhibited the fungal growth in dose dependent manner, resulting in growth induction at low doses. While, pea extract inhibited the FB production in most of the strains (Stȩpień et al., 2015).

Modulation of spore germination is also often based on low-molecular weight substances produced by the plant host. For example, flavonoids stimulated the germination of conidia of *F. solani* on the leaves of vegetables (Ruan et al., 1995). Moreover, Garcia et al. (2012) found that production of FB1 and FB2 by *F. verticillioides*, and ZEA and DON by *F. graminearum* was stimulated or similar to the controls in most of the conditions tested using *Equisetum arvense* and *Stevia rebaudiana* extracts.

### Aspergilli

**Figure 10:**
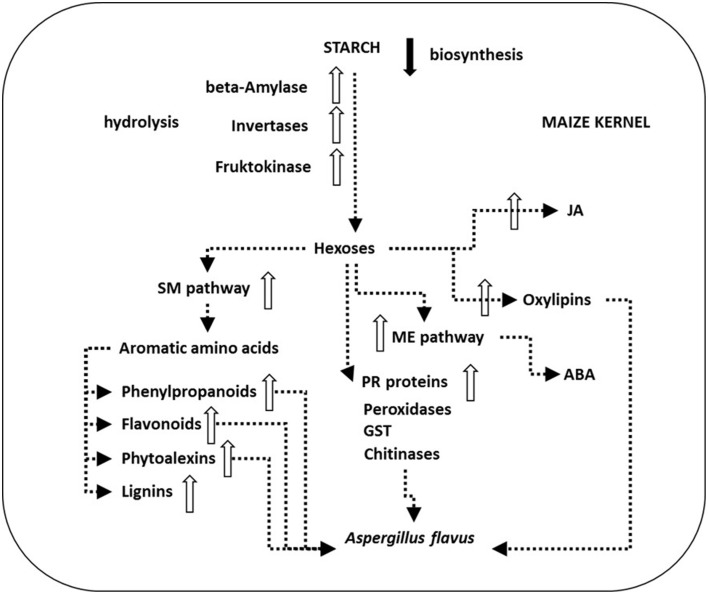
**Schematic presentation of main plant defense processes in ***Aspergillus flavus***-maize interactions**. *A. flavus* can attack kernels during all the six stages of their development. However, infection in non-injured kernels takes place later in the field, during the dent (R5) developmental stage just prior to physiological maturity (R6) (Marsh and Payne, 1984). As soon as 4 days after inoculation *A. flavus* mycelium reaches the aleurone, endosperm and germ tissue (Dolezal et al., 2013). Transcriptional analysis of the maize—*A. flavus* pathogen interaction revealed down-regulated (black arrow) starch biosynthesis and up-regulated genes (white arrows) of plant starch hydrolytic enzymes like β-amylase as well as downstream invertases and fructokinase. The produced hexoses flow through the up-regulated shikimate (SM) pathway, the methylerithryole (ME) pathway and toward up-regulated jasmonic acid (JA) and oxylipin biosynthesis, and feed pathogenesis related (PR) protein synthesis, e.g., peroxidases, glutathione S-transferase (GST) or chitinases that were also found up-regulated during infection. Oxylipins up-regulate aflatoxin (AF) biosynthesis and sexual reproduction in *A. flavus* and down-regulate fungal growth. Up-regulation of the SM pathway leads to the production of antifungal compounds flavonoids, phenylpropanoids, phytoalexins, and up-regulated lignin production in maize. Up-regulated plant hormone JA and abscisic acid (ABA) production is crucial in these defense mechanisms (Dolezal et al., 2014).

*Aspergillus* species can be saprophytic, or symptomless endophytes or weak and opportunistic plant pathogen. *A. flavus* from yellow Aspergilli is a weak and opportunistic plant pathogen. It lacks host specificity (St Leger et al., 2000) as it can attack seeds of both monocots and dicots such as maize, cotton, groundnuts (peanuts) and other nuts like tree nuts such as Brazil nuts, pecans, pistachio nuts, and walnuts. *A. flavus* can cause ear rot on maize and preharvest contamination of these crops with SM AFs is common, but *A. flavus* also causes the spoilage of post-harvest grains during storage resulting in significant economic losses to farmers (Figure 10).

*A. flavus, A. parasiticus* and *A. nidulans* are proposed to derive acetyl CoA for the biosynthesis of SM toxins (i.e., sterigmatocystin and AF) (Figure 5) from fatty acids present in the kernel of maize (Howlett, 2006). *In vitro* supplemented oleic acid induced the biogenesis of fungal peroxisomes, as well as catalase activity and β-oxidation. Concomitantly with the increased expression of biosynthetic genes for precursor sterigmatocystin and AF in hyphae, colonizing the embryo and aleuronic layer, where most seed lipids are stored, AF precursor norsolorinic acid accumulated in peroxisomes (Maggio-Hall et al., 2005).

Bagheri-Gavkosh et al. (2009) showed that AF B1 production by *A. parasiticus* was inhibited by methanolic extracts of *Ephedra major* aerial parts and roots, whereas the essential oil of the plant aerial parts did not exhibit any effect on AF B1 biosynthesis. The authors attributed the inhibition of *A. parasiticus* growth and AF B1 production to the presence of flavonoid compounds such as *p*-coumaric acid and quercetin in plant extracts. Dos Santos and Furlong (2008) noted that AF B1 and AF B2 production by *A. flavus* was inhibited in the presence of methanolic extracts from banana pulp and peel, orange, eggplant and potato pulp. However, these authors found that in the presence of banana pulp and potato pulp extracts, *A. flavus* produced AF B2, which was not detected in the control. Crude essential oil of *Betula alba* also inhibited both AF production and fungal growth in parallel. Jermnak et al. (2012) found that after roughly purifying the oil by silica gel column chromatography an active fraction was obtained that was identified as methyl syringate. This compound strongly inhibited norsolorinic acid production, an early step of the AF biosynthetic pathway: it inhibited the AF B1 and AF G1 production of *A. parasiticus* in liquid medium in a dose-dependent manner and also inhibited AF B1 production by *A. flavus* on raw peanuts.

Black aspergilli are common soil organisms decomposing dead plant residues, some of them are capable of a biotrophic endophytic existence with maize and onion. *A. niger var. niger* and *A. carbonarius* black Aspergilli are the two major producers of ochratoxin A (Figure 4) that is nephrotoxic, teratogenic, carcinogenic, and immunosuppressive in animals, and of FB1 mycotoxin (Palencia et al., 2010).

In *A. terreus* infection, crops such as wheat, ryegrass and potatoes were shown to acquire disease. A number of SMs and mycotoxins, including territrem A, citreoviridin, citrinin, gliotoxin (Figure 4), patulin, terrein, terreic acid, and terretonin are coded in *A. terreus* (Guo and Wang, 2014). The phytotoxic SM terrein possessed ecological, antimicrobial, antiproliferative, and antioxidative activities was also highly induced in plant-derived media and in induced lesions on fruit surfaces (Zaehle et al., 2014).

### *Rhizoctonia solani* species complex

The soil-borne fungus *Rhizoctonia solani* (teleomorph *Thanatephorus cucumeris*), belonging to the phylum *Basidiomycota*, is an economically important plant pathogen. *R. solani*, as a non-obligate necrotrophic pathogen, causes diseases in many crops including species in the families *Solanaceae, Fabaceae, Asteraceae, Poaceae*, and *Brassicaceae* as well as ornamental plants and forest trees throughout the world (Gonzalez Garcia et al., 2006). The anamorph *R. solani* is a species complex and consists species of at least 14 different, genetically isolated populations [=anastomosis groups (AG)] that differ in their ecology and host range (Carling et al., 2002; Stodart et al., 2007).

Recently it was considered that *R. solani* synthesizes HSTs and NHSTs (Vidhyasekaran et al., 1997). HSTs from *R. solani* could increase the virulence of the pathogen (e.g., HC-toxin on maize) (Brooks, 2007), and were often pathogenicity determinants required for a pathogen to incite disease (reviewed in Wolpert et al., 2002). For example, significantly increased five new superoxide dismutase (SOD) activities were detected in plant under treatment of rice leaf sheaths with *R. solani*-toxin, which eliminated the antifungal oxidative burst (Paranidharan et al., 2005).

Previous research has shown that *R. solani* also produces phenyl acetic acid and its derivatives (Bartz et al., 2013), a phenolic compound, and a carbohydrate as phytotoxins that supported the broad host range and diversity within the *R. solani* species. Xu et al. (2015) identified eight compounds from fermentation broth of *R. solani*, from which *m*-hydroxymethylphenyl pentanoate, (Z)-3-methylpent-2-en-1,5-dioic acid and 3-methoxyfuran-2-carboxylic acid showed phytotoxicity *in vitro*.

Interestingly, in the *R. solani* AG1-IA genome project, genes homologous to reported mycotoxin biosynthesis genes could not be identified (Zheng et al., 2013). However, genes for a putative phytotoxin enniatin (Figure 4) and sequences featuring homology to putative trichothecene citrinin, and AF (Figure 4) and terpene biosynthesis genes (e.g., genes encoding sesquiterpene synthases) were identified. In addition, three volvatoxin genes that homologous to volvatoxin from *Volvariella volvacea* (*Basidiomycota*) were also detected (Wibberg et al., 2014). In proteome analysis, a trichothecene 3-O-acetyltransferase that is required for trichothecene biosynthesis and is involved in reducing the toxicity of trichothecene mycotoxin DON of *Fusaria* (Audenaert et al., 2014), was differentially expressed during the development stage in *R. solani* AG1 (Kwon et al., 2014).

In the last decade, newly related chemical structures have been reported to have significant antifungal activity against *R. solani*: arvelexin isolated from *Thlaspi arvense* (stinkweed) (Pedras et al., 2003), isalexin, brassicanate A and rutalexin from *Brassica napus*, ssp. *rapifera* (Pedras et al., 2004). Cauliflower (*Brassica oleracea* var. *botrytis*) produced other phytoalexins caulilexins A, B, and C (Figure 7), which were also active against the economically important pathogenic fungi *Leptosphaeria maculans* and *S. sclerotiorum* (Pedras et al., 2006). Pedras and Ahiahonu (2005) reviewed the detoxification metabolism of phytoalexins in phytopathogenic fungi. Indole-3-acetaldoxime is an intermediate in the biosynthesis of diverse plant SMs such as indole-3-acetonitrile, brassinin, and brassilexin, as well as the indole glucosinolate (glucobrassicin) and the plant hormone IAA (Figure 1) in *Cruciferae*. Metabolism of indole-3-acetaldoxime to IAA *via* indole-3-acetonitrile by fungi could support the development of plant diseases in crucifers (Pedras and Montaut, 2003).

### Genus cochliobolus

The filamentous ascomycete genus *Cochliobolus* (anamorph *Bipolaris*/*Curvularia*; Manamgoda et al., 2014) is composed of more than 40 closely related pathogenic species with particular specificity to their host plants (Condon et al., 2013). Gao et al. (2014) reported the genome sequence of a highly virulent *C. lunatus* strain, and phylogenomic analysis indicated that *C. lunatus* was evolved from *C. heterostrophus*. *C. lunatus* CX-3 strain was capable of producing diverse SMs (Table 1) such as NHSTs and melanin that could aid in niche exploitation and pathogenicity.

It was known previously that the ability to produce HST T-toxin requires three genes encoded at two unlinked loci (Baker et al., 2006). However, Inderbitzin et al. (2010) reported further six genes including two PKSs, one decarboxylase, five dehydrogenases, and one unknown protein that were involved in T-toxin production and high virulence of *C. heterostrophus* to maize. HST1, one NPRS of *C. carbonum* (*Bipolaris zeicola*) played a key role in the cyclic tetrapeptide HC-toxin (Figure 2) biosynthesis (Walton, 2006), which was also produced by *Alternaria jesenskae* (Wight et al., 2013) and was also encoded in another maize pathogen *Setosphaeria turcica* (Condon et al., 2013). Six other known PKSs were found to be involved in different kinds of toxin biosynthesis such as *A. alternata* ACT-toxin, *F. graminearum* ZEA, *F. verticilloides* fumonisin, *A. ochraceus* OTA and *C. heterostrophus* T-toxin. Phylogenetic and modular analyses suggested that the protein structures of *C. lunatus* CX-3 NRPSs were obviously different from other known NRPSs being involved in the biosynthesis of mycotoxins such as HC-toxin (Figure 2) of *C. carbonum*, similarly to AM-toxin (Figure 2) of *A. alternata*, gliotoxin (Figure 4) of *A. fumigatus* and enniatin of *F. equiseti* (Gao et al., 2014).

### Genus alternaria

*Alternaria* species have different lifestyles ranging from saprophytes to endophytes and to pathogens. Phylogenetic relations of the *Alternaria* complex was revisited and delineated by Woudenberg et al. (2013) within *Alternaria* and related genera based on nucleotide sequence data. *A. alternata* has the ability to produce more than 60 SMs from which at least 10 PKS products can be found (Saha et al., 2012) (Table 1). *Alternaria* species have been reported to cause diseases in nearly 400 plant species including a wide variety of economically important crops and cause severe economic problems. *A. alternata* alone can infect more than 100 plant species (Thomma, 2003). The production of diverse phytotoxins and HSTs can be considered as a key reason for the success of these pathogens (Nishimura and Kohmoto, 1983). From about 20 HSTs that have been documented (Otani et al., 1995; Walton, 1996), at least seven are from *A. alternata* pathotypes (Otani et al., 1995). For HST ACR-toxin production and pathogenicity, PKS gene ACRTS2 was found to be essential of the rough lemon pathotype of *A. alternata* (Izumi et al., 2012). Several NHSTs are also produced in *Alternaria* such as brefeldin A, altertoxin, and tentoxin (Figure 2) and also other mycotoxins. Alternariol (Figure 2) and alternariol-9-methyl ether are major NHSTs that are common contaminants of food such as cereals, fruits and fruit juices (Scott, 2001). A PKS involved in melanin biosynthesis was also characterized and named ALM (albino) (Kimura and Tsuge, 1993). For production of SM siderophores and virulence, the *A. alternata* gene AaNPS6, encoding a polypeptide analogous to fungal NRPS was demonstrated by Chen et al. (2013). The *Alternaria* toxins provide prospect for biocontrol of weeds due to high phytotoxic effect against weeds but low mammalian toxicity (Abbas et al., 1995; Chen et al., 2005; Evidente et al., 2009; Yang et al., 2012).

From the plant side, high concentrations of alkaloid phytoalexin camalexin (Tsuji et al., 1992) have been observed at the infection site of *A. alternata* (Schuhegger et al., 2007) and also in the proximity to the lesions induced by *Botrytis* species (Kliebenstein et al., 2005). In *A. thaliana* leaves both biotrophic and necrotrophic plant pathogens induced camalexin formation (Thomma et al., 1999). *A. brassicicola* could detoxify camalexin but at much slower rate than phytoalexin brassinin from *Brassicaceae* (Pedras et al., 2014).

### Secondary metabolite production of biotrophs

Growth and reproduction of obligate biotrophic phytopathogens that are very poor in SM production like powdery mildews are entirely dependent on living plant cells. Spanu et al. (2010) hypothesized that *Blumeria* synthesized only one iron siderophore and one simple polyketide pigment of the cleistothecia. Similar trends have been observed in other biotrophs, such as the basidiomycete corn smut fungus *Ustilago maydis* and the plant symbiotic fungus *Tuber melanosporum*.

*Cladosporium fulvum* (*Passalora fulva*) is also a biotrophic fungus that infecting tomato, grows extracellularly in close contact with host mesophyll cells. The only known SMs produced by *C. fulvum* is cladofulvin (de Wit et al., 2012; Collemare et al., 2014) anthraquinone pigment. However, cladofulvin has not been detected to cause necrosis on *Solanaceae* plants or to show any antimicrobial activity (Collemare et al., 2014). *C. fulvum* has also the potential to produce elsinochrome and cercosporin toxins, but the corresponding core genes were not expressed during infection of tomato (Collemare et al., 2014). It has been suggested that loss of SM biosynthetic pathways is associated with biotrophy (Spanu et al., 2010); nevertheless, the biotrophic *C. fulvum* has twice the number of key SM genes compared to the closely related hemibiotrophic *Dothistroma septosporum* (teleomorph *Mycosphaerella pini*), of which 14 and 9, respectively, are organized into gene clusters along with other SM-related genes (de Wit et al., 2012). The numbers of its SM enzyme-encoding genes were comparable to those of *M. graminicola*, but were lower than those in most other sequenced Dothideomycete (Table 1). It could be concluded that, in contrast to reduced SM production capacity, down-regulation of high number of SM biosynthetic pathways might represent another mechanism associated with a biotrophic lifestyle (Collemare et al., 2014).

## Conclusions and future aspects

Fungal-plant host interactions represent biochemically complex and challenging scenarios that are being investigated also by metabolomic approaches (Allwood et al., 2008). It is noteworthy that although SMs play important roles in the virulence and lifestyle of fungal plant pathogens only about 25% of the fungal SM gene clusters have already been characterized functionally and this number is much lower at plant side.

Concomitantly, comparative genomics and transcriptomics are employed to obtain insights into the genetic features that enable fungal pathogens to adapt successfully to various ecological niches and to adopt different pathogenic lifestyles. The suites of fungal SM genes reflect astounding diversity among species, hinting that gene products, particularly those associated with unique genomic regions, are candidates for pathogenic lifestyle differences. Furthermore, horizontal gene and chromosome transfers provide a means for pathogens to broaden their host range (Mehrabi et al., 2011; Fitzpatrick, 2012). The increasing availability of fungal pathogen genome sequences and next-generation genomic tools allow us to survey the SM gene clusters in individual fungi. The recent availability of next-generation RNA-Seq technologies has revolutionized transcriptomic profiling and are used to probe the expression of SM gene clusters during various stages of infection. Unlike microarray, RNA-Seq allows the simultaneous quantification of transcripts from more than one organism and is thus perfectly suited for the study of plant-pathogen interactions (Chooi and Solomon, 2014). Moreover, manipulations of strain-unique SM genes associated with host-specific virulence provide possibility to investigate fungal-plant interaction.

The great structural diversity of phytotoxins, the high potency and exclusive mechanisms of action (compared to synthetic herbicides) make fungal toxins highly attractive for discovering herbicidal activity. Even if natural phytotoxins are not necessarily suitable for direct use as a commercial herbicide, the identification of mechanisms are very important for new herbicide developments. Newly developed herbicides with environmentally friendly component could be used more safely in integrated pest management systems. On the other side, plant SMs can be used against plant pathogens (especially in sprayable forms) as natural plant extracts for example in organic agricultural production systems. Deeper knowledge of the fungus-plant interaction may help resistance breeding of new plant cultivars/hybrids against stresses such as abiotic (e.g., heat) stress or against fungal diseases.

### Conflict of interest statement

The authors declare that the research was conducted in the absence of any commercial or financial relationships that could be construed as a potential conflict of interest.

## Abbreviations

ABA: abscisic acid
AF: aflatoxin
AF B1: aflatoxin B1
AF B2: aflatoxin B2
AF G1: aflatoxin G1
DMATS: dimethylallyl tryptophan synthetase
DON: deoxynivalenol
ET: ethylene
FB1: fumonisin B1
FB2: fumonisin B2
JA: jasmonic acid
IAA: indole-3-acetic acid
ISR: induced systemic resistance
HST: host-selective toxin
NHST: non host-selective toxin
NRPS: non-ribosomal protein synthase
PCD: programmed cell death
PKS: polyketide synthase
PR: pathogenesis-related
ROS: reactive oxygen species
SA: salicylic acid
SAR: systemic acquired resistance
SM: secondary metabolite
TS: tryptophan synthetase
ZEA: zearalenone.
